# Depression in patients with inflammatory bowel disease is associated with increased risk of dementia and Parkinson’s disease: A nationwide, population-based study

**DOI:** 10.3389/fmed.2022.1014290

**Published:** 2022-10-06

**Authors:** Kookhwan Choi, Hyun Jung Lee, Kyungdo Han, Seong-Joon Koh, Jong Pil Im, Joo Sung Kim

**Affiliations:** ^1^Department of Internal Medicine and Liver Research Institute, Seoul National University College of Medicine, Seoul, South Korea; ^2^Department of Statistics and Actuarial Science, Soongsil University, Seoul, South Korea

**Keywords:** dementia, Parkinson’s disease, depression, inflammatory bowel disease, claims data

## Abstract

**Background:**

Inflammatory bowel disease (IBD) may be associated with depression which is considered an important cause of dementia and Parkinson’s disease (PD). In the present study, the effects of depression on the development of dementia and/or PD in patients with IBD were evaluated.

**Materials and methods:**

A nationwide population-based cohort study was conducted using claims data from the Health Insurance Review and Assessment Service in Korea. The incidence of dementia and PD were analyzed based on the presence of depression in patients with IBD.

**Results:**

During a mean follow-up of 8 years, IBD patients with depression experienced dementia (6.7 vs. 2.0%; *p* < 0.001) and PD (1.1 vs. 0.3%; *p* < 0.001) significantly more than IBD patients without depression. Compared with IBD patients without depression, the risk of developing dementia was significantly higher in IBD patients with depression [adjusted hazard ratio (aHR) for IBD, Crohn’s disease (CD), and ulcerative colitis (UC), 2.03, *p* < 0.001; 1.68, *p* = 0.033; 2.13, *p* < 0.001, respectively]. Compared with IBD patients without depression, the risk of developing PD was significantly higher in IBD patients with depression (aHR for IBD, CD, and UC, 2.54, *p* < 0.001; 1.93, *p* = 0.470; 2.75, *p* < 0.001, respectively). The cumulative incidence of dementia and PD in IBD patients with depression was significantly higher than in IBD patients without depression and showed a steady increase after a diagnosis of depression.

**Conclusion:**

The risk of dementia and/or PD increased after a diagnosis of depression in patients with IBD.

## Introduction

Dementia and Parkinson’s disease (PD) are the most common neurodegenerative disorders characterized by chronic progressive disease that may lead to death (1, 2). The prevalence of dementia and PD and the resulting economic burden have increased rapidly over the years in South Korea (3, 4). Recently, numerous evidence has shown that systemic immune activation affects development of dementia and PD, indicating chronic inflammatory conditions in peripheral organs may be associated with neurodegeneration in dementia and PD by altering blood-brain barrier function (5, 6). In addition, activation of microglia was observed around the cranial nerves, and the levels of peripheral inflammatory cytokines, such as interleukin (IL)-6, tumor necrosis factor (TNF), IL-1, IL-10, and C-reactive protein, were increased in both dementia and PD patients (7, 8).

Inflammatory bowel disease (IBD), such as Crohn’s disease (CD) and ulcerative colitis (UC), are immune-related, and chronic inflammatory diseases may play a role in development of neurodegenerative disorders through chronic systemic inflammation and disruption of intestinal and blood-brain barriers (9, 10). Furthermore, the homeostasis of the gut microbiome is an important factor in regulating the brain-gut axis, however, dysbiosis is frequently observed in IBD patients and may cause psychological disorders (11). In several recent nationwide studies, overall incidence of dementia among patients with IBD was significantly increased compared with controls (5.5 vs. 1.4%) and the risk of developing PD in patients with IBD was significantly higher than in the general population (adjusted hazard ratio (aHR), 1.87) (12, 13).

Similarly, the risk of developing depression increases in patients with IBD (14), and depression is an important cause of dementia and PD (15, 16). However, the relationship between the presence of depression and development of dementia and/or PD in patients with IBD has not been elucidated. In this nationwide population-based study, the effects of depression on developing dementia and/or PD in patients with IBD were investigated.

## Materials and methods

### Data source

The present population-based cohort study was performed using data from the Health Insurance Review and Assessment Service (HIRA), which is the nationwide mandatory health care system that covers approximately 97% of healthcare service providers and citizens in South Korea (17). The HIRA database includes information on demographics, disease codes, prescription codes, procedural codes, as well as outpatient and inpatient care records. These data are processed with non-identifiable codes to avoid recognition of personal information. The HIRA established a registration program in 2006 for rare intractable diseases (RIDs), such as IBD, and patients must register in the RID program to receive co-payment reduction. The RID codes were defined by the International Classification of Diseases, tenth revision (ICD-10) code and a special code (V code) recorded in the RID database. The RID code is highly reliable because the patient must satisfy the diagnostic criteria for the specific RID and validated by a qualified physician (18). The accuracy of the incident IBD assigned by both V and ICD-10 codes was proven in previous studies (12, 14). Furthermore, sensitivity analysis was performed from January 2010 to March 2013 at Seoul National University Hospital, a tertiary referral hospital in Korea, and the diagnostic sensitivity of CD and UC was 94.5 and 96.4%, respectively.

### Study population

From January 2010 to December 2017, all patients with IBD assigned both the ICD-10 (CD, K50; UC, K51) and V codes (CD, V130; UC, V131) were included in the present study. The study participants were divided into the following two groups: the incident group, defined as patients with IBD who were newly diagnosed with depression between January 2010 and December 2017, and the prevalent group, defined as patients previously diagnosed with depression and coded with IBD between January 2010 and December 2017. In this study, the incident group was analyzed separately from the prevalent group to determine whether newly developed depression in IBD patients increased the risk of dementia and/or Parkinson’s disease.

Depression was detected using ICD-10 codes (F32–34) as defined in previous studies (12, 14). A washout period of 7 years (from January 2004 to December 2010) was included and individuals with missing data for any variable were excluded.

### Data collection

Demographic data of the study population (age, sex, residence), comorbidities, and medication use for IBD were collected. Information regarding comorbidities, including hypertension (ICD-10 codes (I10–13, I15) treated with anti-hypertensive agents or systolic/diastolic blood pressure ≥ 140/90 mmHg), diabetes mellitus [DM, ICD-10 codes (E11–14) treated with anti-hyperglycemic agents or fasting glucose level ≥ 126 mg/dl], dyslipidemia [ICD-10 code (E78) treated with anti-hyperlipidemic agents or fasting total cholesterol ≥ 240 mg/dl], myocardial infarction (ICD-10 codes (I21–22) with hospitalization) or stroke (ICD-10 codes (I63–64) with hospitalization and brain imaging using computed tomography or magnetic resonance imaging) were defined using ICD-10 codes as defined in previous studies (19, 20). In addition, information regarding therapeutic drug use for IBD, including 5-aminosalicylic acid (5-ASA), immunomodulators (azathioprine, 6-mercaptopurine, and methotrexate), steroids, biologics (infliximab, adalimumab, golimumab, and vedolizumab), and small molecules (tofacitinib), within 1 year of IBD diagnosis was collected.

### Study endpoint

The primary outcome was newly diagnosed dementia and/or PD after the onset of depression in patients with IBD. Alzheimer’s dementia was diagnosed with ICD-10 codes (F00 and G30) and vascular dementia with F01 code, as defined in a previous study (21, 22). Patients with PD were assigned both ICD-10 (G20) and V codes (V124) as defined in previous studies (12, 23). All patients with IBD who had a past history of dementia and/or PD were excluded to only include individuals who experienced an initial onset of dementia and/or PD following the presentation of depression in patients with IBD. All patients were followed up until the occurrence of dementia and/or PD or December 2017.

### Statistical analysis

To compare characteristics between two groups, *t*-tests were used for continuous variables and chi-square tests were used for categorical variables. Continuous variables were presented as means ± standard deviation (SD) and categorical variables as number and percentage. Cox proportional hazards regression models were used to assess the risk of newly developed dementia and/or PD associated with depression in patients with IBD. Competing risk analysis was performed using the Fine-Gray Competing risk model. Results were expressed as adjusted hazard ratio (aHR) and 95% confidence interval (CI). The potential modification effect based on age, sex, residence, comorbidities, and therapeutic drug use for IBD was evaluated through stratified analysis and interaction testing using a forest plot. The cumulative incidence for each group was plotted with Kaplan-Meier curves and compared using the log-rank test. All statistical tests were two-tailed and the statistical significance level was set at a *p* < 0.05. Statistical analyses were performed using R programming version 3.4.3 (The R Foundation for Statistical Computing, Vienna, Austria^1^) and SAS Version 9.2 (SAS Institute Inc., Cary, NC, USA) for Windows.

## Results

### Baseline characteristics of the study population

From January 2010 to December 2017, a total of 17,391 IBD patients were enrolled in the present study; 421 individuals (2.42%) developed dementia and 64 (0.37%) developed PD during a median follow-up of 8 years. The baseline characteristics of the study population based on the occurrence of dementia and PD are shown in Tables 1, 2, respectively. Regarding development of dementia in IBD patients with or without depression, there were 421 subjects with dementia and 16,970 without dementia (Table 1). Patients with dementia were more likely to be older (*p* < 0.001), female (*p* < 0.001), and have a rural residence (*p* < 0.001). The prevalence of comorbidities including hypertension, DM, dyslipidemia, and stroke was significantly higher in patients with dementia than patients without dementia (*p* < 0.001 for each variable). However, therapeutic drug use for IBD, including 5-ASA and immunomodulators, was significantly more frequent in patients without dementia than in patients with dementia (*p* < 0.05 for each drug).

**TABLE 1:** Baseline characteristics of the study population in development of dementia on inflammatory bowel disease (IBD).

	IBD			CD			UC		
No. (%)	Non-dementia	Dementia	*P*-value	Non-dementia	Dementia	*P*-value	Non-dementia	Dementia	*P*-value
	16,970	421		2,729	90		14,241	331	
Male gender	10,258 (60.45)	197 (46.79)	<0.001	1,561 (57.2)	38 (42.22)	0.005	8,697 (61.07)	159 (48.04)	<0.001
Age, years^†^	54.21 ± 10.07	70.47 ± 9.42	<0.001	53.93 ± 10.65	70.81 ± 9.23	<0.001	54.26 ± 9.96	70.38 ± 9.48	<0.001
≤40	734 (4.33)	1 (0.24)		159 (5.83)	0 (0)		575 (4.04)	1 (0.3)	
41–60	11,900 (70.12)	68 (16.15)		1,877 (68.78)	12 (13.33)		10,023 (70.38)	56 (16.92)	
60<	4,336 (25.55)	352 (83.61)		693 (25.39)	78 (86.67)		3,643 (25.58)	274 (82.78)	
Rural residence	6,676 (39.34)	206 (48.93)	<0.001	1,002 (36.72)	46 (51.11)	0.005	5,674 (39.84)	160 (48.34)	0.002
**Comorbidities**									
Diabetes mellitus	1,430 (8.43)	99 (23.52)	<0.001	287 (10.52)	0	<0.001	1,143 (8.03)	78 (23.56)	<0.001
Hypertension	4,318 (25.44)	232 (55.11)	<0.001	737 (27.01)	54 (60)	<0.001	3,581 (25.15)	178 (53.78)	<0.001
Dyslipidemia	2,932 (17.28)	128 (30.4)	<0.001	468 (17.15)	31 (34.44)	<0.001	2,464 (17.3)	97 (29.31)	<0.001
Stroke	407 (2.4)	48 (11.4)	<0.001	92 (3.37)	15 (16.67)	<0.001	315 (2.21)	33 (9.97)	<0.001
Myocardial infarction	140 (0.82)	7 (1.66)	0.064	36 (1.32)	2 (2.22)	0.465	104 (0.73)	5 (1.51)	0.103
**Medications for IBD**									
5-ASA	14,589 (85.97)	294 (69.83)	<0.001	2,012 (73.73)	50 (55.56)	<0.001	12,577 (88.32)	244 (73.72)	<0.001
Immunomodulators	2,516 (14.83)	45 (10.69)	0.018	982 (35.98)	18 (20)	0.002	1,534 (10.77)	27 (8.16)	0.128
Steroid	10,230 (60.28)	273 (64.85)	0.059	1,663 (60.94)	58 (64.44)	0.502	8,567 (60.16)	215 (64.95)	0.078
Biologics and small molecules	619 (3.65)	8 (1.9)	0.058	209 (7.66)	3 (3.33)	0.126	410 (2.88)	5 (1.51)	0.139

^†^Mean ± SD; IBD, inflammatory bowel disease; CD, Crohn’s disease; UC, ulcerative colitis.

**TABLE 2:** Baseline characteristics of the study population in development of Parkinson’s disease on inflammatory bowel disease (IBD).

	IBD			CD			UC		
No. (%)	Non-PD	PD	*P*-value	Non-PD	PD	*P*-value	Non-PD	PD	*P*-value
	17,327	64		2,811	8		14,516	56	
Male gender	10,416 (60.11)	39 (60.94)	0.893	1,594 (56.71)	5 (62.5)	0.741	8,822 (60.77)	34 (60.71)	0.993
Age, years^†^	54.56 ± 10.34	66.52 ± 9.87	<0.001	54.45 ± 11.01	60.25 ± 10.57	0.137	54.58 ± 10.21	67.41 ± 9.53	<0.001
≤40	735 (4.24)	0 (0)		159 (5.66)	0 (0)		576 (3.97)	0 (0)	
41–60	11,955 (69)	13 (20.31)		1,885 (67.06)	4 (50)		10,070 (69.37)	9 (16.07)	
60<	4,637 (26.76)	51 (79.69)		767 (27.29)	4 (50)		3,870 (26.66)	47 (83.93)	
Rural residence	6,862 (39.6)	20 (31.25)	0.173	1,047 (37.25)	1 (12.5)	0.148	5,815 (40.06)	19 (33.93)	0.350
**Comorbidities**									
Diabetes mellitus	1,521 (8.78)	8 (12.5)	0.294	307 (10.92)	1 (12.5)	0.886	1,214 (8.36)	7 (12.5)	0.265
Hypertension	4,521 (26.09)	29 (45.31)	<0.001	789 (28.07)	2 (25)	0.847	3,732 (25.71)	27 (48.21)	<0.001
Dyslipidemia	3,037 (17.53)	23 (35.94)	<0.001	494 (17.57)	5 (62.5)	<0.001	2,543 (17.52)	18 (32.14)	0.004
Stroke	451 (2.6)	4 (6.25)	0.068	106 (3.77)	1 (12.5)	0.197	345 (2.38)	3 (5.36)	0.145
Myocardial infarction	146 (0.84)	1 (1.56)	0.53	37 (1.32)	1 (12.5)	0.006	109 (0.75)	0 (0)	0.515
**Medications for IBD**									
5-ASA	14,823 (85.55)	60 (93.75)	0.062	2,055 (73.11)	7 (87.5)	0.359	12,768 (87.96)	53 (94.64)	0.125
Immunomodulators	2,558 (14.76)	3 (4.69)	0.023	999 (35.54)	1 (12.5)	0.174	1,559 (10.74)	2 (3.57)	0.083
Steroid	10,459 (60.36)	44 (68.75)	0.171	1,716 (61.05)	5 (62.5)	0.933	8,743 (60.23)	39 (69.64)	0.151
Biologics and small molecules	626 (3.61)	1 (1.56)	0.38	212 (7.54)	0 (0)	0.419	414 (2.85)	1 (1.79)	0.632

^†^Mean ± SD; IBD, inflammatory bowel disease; CD, Crohn’s disease; UC, ulcerative colitis; PD, Parkinson’s disease.

Regarding the development of PD in IBD patients with or without depression, there were 64 subjects with PD and 17,327 without PD (Table 2). IBD patients with PD were more likely to be older (*p* < 0.001) and have higher prevalence of comorbidities including hypertension and dyslipidemia (*p* < 0.001 for each variable). However, IBD patients without PD used immunomodulators more frequently than patients with PD (*p* = 0.023).

### Incidence and risk of dementia based on presence of depression in patients with inflammatory bowel disease

During the follow-up period, 104 IBD patients (6.7%) with depression and 317 IBD patients (2.0%) without depression developed dementia (*p* < 0.001). The incidence and risk of dementia in the IBD with depression were significantly higher than in the IBD without depression after adjusting for age, sex, residence, comorbidities (e.g., hypertension, DM, dyslipidemia, myocardial infarction, and stroke) and therapeutic drug use for IBD (e.g., 5-ASA, immunomodulators, steroids, biologics, and small molecules; 16.1 vs. 4.4 per 1,000 person-years; aHR, 2.03; 95% CI, 1.62–2.55; *p* < 0.001 in Model 3). When the depression cohort was divided into the incident and prevalent groups, the incidence and risk of dementia were also higher in the IBD with depression than in the IBD without depression (incident group: 12.0 vs. 4.4 per 1,000 person-years; aHR, 1.70; 95% CI, 1.24–2.35; prevalent group: 21.7 vs. 4.4 per 1,000 person-years; aHR, 2.37; 95% CI, 1.79–3.15; all *p* < 0.001 in Model 3). Further sensitivity analysis was conducted for the competing risk of stroke and myocardial infarction and the increased risk dementia in IBD patients with depression remain robust (aHR, 1.87; 95% CI, 1.43–2.43; *p* < 0.001; data not shown).

When further analyses were performed across IBD subtypes, the associations between IBD with depression and the risk of dementia remained robust. In the CD patients with depression, the incidence and risk of dementia were significantly higher than in CD patients without depression (17.2 vs. 6.0 per 1,000 person-years; aHR, 1.68; 95% CI, 1.04–2.71; *p* = 0.033 in Model 3). Similar results were also found between the incident and prevalent groups but without statistical significance in the incident group (incident group: 9.3 vs. 6.0 per 1,000 person-years; aHR, 1.14; 95% CI, 0.52–2.53; *p* = 0.740; prevalent group: 26.6 vs. 6.0 per 1,000 person-years; aHR, 2.10; 95% CI, 1.21–3.65; *p* = 0.009 in Model 3). In UC patients with depression, the incidence and risk of dementia were also higher than in UC patients without depression (15.8 vs. 4.1 per 1,000 person-years; aHR, 2.13; 95% CI, 1.64–2.76; *p* < 0.001 in Model 3). The incidence and risk of dementia showed similar results in both the incident and prevalent groups (incident group: 12.7 vs. 4.1 per 1,000 person-years; aHR, 1.90; 95% CI, 1.34–2.69; prevalent group: 20.3 vs. 4.1 per 1,000 person-years; aHR, 2.39; 95% CI, 1.71–3.33; all *p* < 0.001 in Model 3; Table 3).

**TABLE 3:** Independent risk of dementia according to presence of depression in patients with inflammatory bowel disease (IBD).

	Total no.	Events (*n*)	Follow-up duration (person-years)	Incidence rate (per 1,000 person-years)	Model 1^†^ HR (95% C.L.)	*P*-value	Model 2^‡^ HR (95% C.L.)	*P*-value	Model 3^§^ HR (95% C.L.)	*P*-value
IBD						<0.001		<0.001		<0.001
Without depression	15,843	317	71,751	4.42	1 (Ref.)		1 (Ref.)		1 (Ref.)	
With depression	1,548	104	6,441	16.14	2.28 (1.82–2.85)		2.10 (1.67–2.63)		2.03 (1.62–2.55)	
**Subgroup**										
Incident	849	44	3,679	11.95	1.96 (1.43–2.69)	<0.001	1.77 (1.29–2.44)	<0.001	1.70 (1.24–2.34)	<0.001
Prevalent	699	60	2,762	21.71	2.58 (1.95–3.41)	<0.001	2.43 (1.83–3.22)	<0.001	2.37 (1.79–3.15)	<0.001
CD						0.006		0.033		0.033
Without depression	2,482	66	10,978	6.01	1 (Ref.)		1 (Ref.)		1 (Ref.)	
With depression	337	24	1,393	17.22	1.93 (1.21–3.09)		1.68 (1.04–2.71)		1.68 (1.04–2.71)	
**Subgroup**										
Incident	168	7	754	9.28	1.44 (0.66–3.14)	0.363	1.18 (0.54–2.61)	0.677	1.14 (0.52–2.53)	0.740
Prevalent	169	17	639	26.59	2.25 (1.32–3.85)	0.003	2.04 (1.18–3.56)	0.011	2.1 (1.21–3.64)	0.009
UC						<0.001		<0.001		<0.001
Without depression	13,361	251	60,772	4.13	1 (Ref.)		1 (Ref.)		1 (Ref.)	
With depression	1,211	80	5,048	15.85	2.34 (1.81–3.01)		2.20 (1.70–2.84)		2.13 (1.21–3.65)	
**Subgroup**										
Incident	681	37	2,924	12.65	2.11 (1.49–2.98)	<0.001	1.97 (1.39–2.79)	<0.001	1.90 (1.34–2.69)	<0.001
Prevalent	530	43	2,124	20.25	2.58 (1.86–3.58)	<0.001	2.45 (1.76–3.42)	<0.001	2.39 (1.71–3.33)	<0.001

IBD, inflammatory bowel disease; CD, Crohn’s disease; UC, ulcerative colitis.

^†^Model 1: adjusted for age, sex. ^‡^Model 2: adjusted for model 1 + residence, diabetes mellitus, hypertension, dyslipidemia, myocardial infarction and stroke.

^§^Model 3: adjusted for model 2 + medication use for IBD (5-Aminosalicylic acid, immunomodulators, steroid, biologics and small molecule).

**FIGURE 1:**
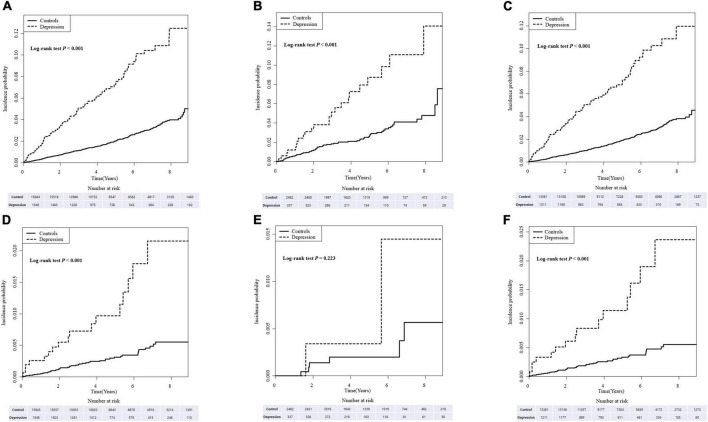
The cumulative incidence of dementia **(A–C)** and Parkinson’s disease (PD) **(D–F)** in inflammatory bowel disease (IBD), Crohn’s disease (CD), and ulcerative colitis (UC) patients with depression compared with those without depression.

The cumulative incidence of dementia in IBD, CD, and UC patients with depression was significantly higher than in IBD, CD, and UC patients without depression (all *p* < 0.001; Figures 1A–C). Similarly, when dementia was divided into Alzheimer’s dementia and vascular dementia subgroups, a significant increase in the incidence and risk of dementia was found in IBD, CD, and UC patients with depression compared with IBD, CD, and UC patients without depression. However, the difference in incidence of vascular dementia in CD patients was statistically non-significant (*p* = 0.460; Supplementary Tables 1, 2 and Supplementary Figures 1A–F).

### Incidence and risk of Parkinson’s disease based on the presence of depression in patients with inflammatory bowel disease

During the follow-up period, 17 IBD patients (1.1%) with depression and 47 IBD patients (0.3%) without depression developed PD (*p* < 0.001). The incidence and risk of PD in the IBD with depression were significantly higher than in the IBD without depression after adjusting for age, sex, place of residence, comorbidities, and therapeutic drug use for IBD (2.5 vs. 0.6 per 1,000 person-years; aHR, 2.54; 95% CI, 1.43–4.51; *p* < 0.001 in Model 3). Similar results were also found between the incident and prevalent groups, however, statistical significance was not observed in the incident group (incident group: 1.3 vs. 0.6 per 1,000 person-years; aHR, 1.53; 95% CI, 0.60–3.90; *p* = 0.368; prevalent group: 4.2 vs. 0.6 per 1,000 person-years; aHR, 3.55; 95% CI, 1.84–6.88; *p* < 0.001 in Model 3). Further sensitivity analysis for the competing risk of stroke and myocardial infarction also confirmed the robustness of main results (aHR, 2.37; 95% CI, 1.27–4.4; *p* = 0.006; data not shown).

In patients with CD, the incidence and risk of PD was higher in patients with depression but without statistical significance (1.4 vs. 0.5 per 1,000 person-years; aHR, 1.93; 95% CI, 0.32–11.59; *p* = 0.470 in Model 3). Further analyses based on incident and prevalent depression showed similar results that incidence and risk of PD was also higher in the CD patients with depression than in CD patients without depression, however, statistical significance was not observed (incident group: 1.3 vs. 0.5 per 1,000 person-years; aHR, 2.85; 95% CI, 0.33–24.74; *p* = 0.342; prevalent group: 1.5 vs. 0.5 per 1,000 person-years; aHR, 1.27; 95% CI, 0.10–16.51; all *p* = 0.857 in Model 3). The incidence and risk of PD were higher in UC patients with depression than in UC patients without depression (2.9 vs. 0.7 per 1,000 person-years; aHR, 2.75; 95% CI, 1.50–5.04; *p* < 0.001 in Model 3) which was similar to the incident and prevalent groups. However, statistical significance was not observed in the incident group (incident group: 1.3 vs. 0.7 per 1,000 person-years; aHR, 1.38; 95% CI, 0.49–3.89; *p* = 0.541; prevalent group: 5.0 vs. 0.7 per 1,000 person-years; aHR, 4.34; 95% CI, 2.18–8.64; *p* < 0.001 in Model 3; Table 4).

**TABLE 4:** Independent risk of Parkinson’s disease according to presence of depression in patients with inflammatory bowel disease (IBD).

	Total no.	Events (*n*)	Follow-up duration (person-years)	Incidence rate (per 1,000 person-years)	Model 1^†^ HR (95% C.L.)	*P*-value	Model 2^‡^ HR (95% C.L.)	*P*-value	Model 3^§^ HR (95% C.L.)	*P*-value
IBD						<0.001		<0.001		<0.001
Without depression	15,843	47	72,355	0.65	1 (Ref.)		1 (Ref.)		1 (Ref.)	
With depression	1,548	17	6,660	2.55	2.68 (1.53–4.71)		2.53 (1.43–4.47)		2.54 (1.43–4.51)	
**Subgroup**										
Incident	849	5	3,786	1.32	1.56 (0.62–3.94)	0.344	1.53 (0.61–3.86)	0.371	1.53 (0.61–3.89)	0.368
Prevalent	699	12	2,874	4.17	3.87 (2.03–7.38)	<0.001	3.53 (1.83–6.83)	<0.001	3.55 (1.84–6.88)	<0.001
CD						0.343		0.417		0.470
Without depression	2,482	6	11,107	0.54	1 (Ref.)		1 (Ref.)		1 (Ref.)	
With depression	337	2	1,437	1.39	2.19 (0.43–11.11)		2.09 (0.35–12.38)		1.93 (0.32–11.59)	
**Subgroup**										
Incident	168	1	767	1.30	2.39 (0.29–20.07)	0.421	3.09 (0.36–26.86)	0.306	2.85 (0.33–24.74)	0.342
Prevalent	169	1	670	1.49	2.01 (0.23–17.43)	0.524	1.40 (0.12–16.96)	0.790	1.27 (0.09–16.51)	0.857
UC						<0.001		<0.001		<0.001
Without depression	13,361	41	61,247	0.67	1 (Ref.)		1 (Ref.)		1 (Ref.)	
With depression	1,211	15	5,223	2.87	2.78 (1531–5.07)		2.73 (1.49–5.00)		2.75 (1.50–5.04)	
**Subgroup**										
Incident	681	4	3,019	1.32	1.41 (0.51–3.96)	0.510	1.39 (0.49–3.90)	0.534	1.38 (0.49–3.89)	0.541
Prevalent	530	11	2,204	4.99	4.36 (2.21–8.59)	<0.001	4.29 (2.16–8.55)	<0.001	4.34 (2.18–8.64)	<0.001

IBD, inflammatory bowel disease; CD, Crohn’s disease; UC, ulcerative colitis.

^†^Model 1: adjusted for age, sex. ^‡^Model 2: adjusted for model 1 + residence, diabetes mellitus, hypertension, dyslipidemia, myocardial infarction and stroke.

^§^Model 3: adjusted for model 2 + medication use for IBD (5-Aminosalicylic acid, immunomodulators, steroid, biologics and small molecule).

The cumulative incidence of PD in the IBD, CD, and UC patients with depression was significantly higher compared with IBD, CD, and UC patients without depression. However, statistical significance was not observed in CD patients with depression (Figures 1D–F).

### Subgroup analysis

**FIGURE 2:**
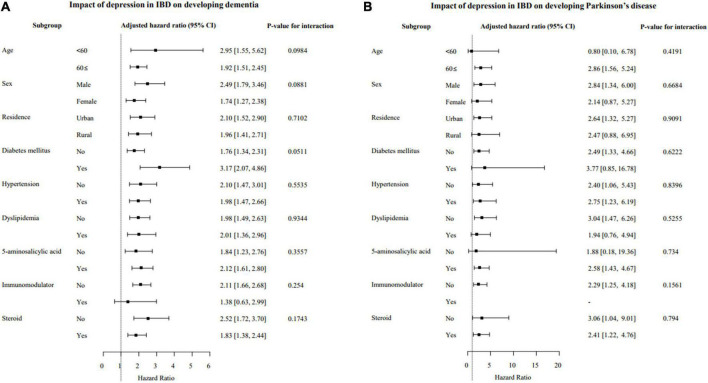
Subgroup analysis of the risk of developing dementia **(A)** and Parkinson’s disease (PD) **(B)** based on the presence of depression in patients with IBD. IBD, inflammatory bowel disease; CI, confidence interval.

In subgroup analysis, differences in the risk of developing dementia and/or PD based on age, sex, comorbidities (hypertension, DM, dyslipidemia), and therapeutic drug use for IBD (5-ASA, immunomodulators and steroids) were evaluated. The effects of depression in IBD patients on developing dementia and/or PD were not significantly different based on subgroups indicating depression independently increased the incidence of dementia and/or PD regardless of the subgroup (Figures 2A,B). However, the effects of depression in IBD patients on developing Alzheimer’s dementia were significantly greater in patients with DM (aHR, 3.56 vs. 1.64; interaction *p* = 0.013; Supplementary Figures 2A,B).

## Discussion

In this nationwide, population-based cohort study including 17,391 patients with IBD in the HIRA database, a significantly increased risk of developing dementia and/or PD in IBD patients with depression was found compared with IBD patients without depression. IBD patients with depression were 2.0- and 2.5-fold more likely to develop dementia and PD, respectively, compared with IBD patients without depression, even after adjusting for age, sex, residence, comorbidities, and therapeutic drug use for IBD.

The effects of depression on developing dementia and/or PD were consistent across IBD subtypes and the cumulative incidence showed a steady increase for 8 years after diagnosis of IBD with depression. The effects of depression on the risk of developing dementia and/or PD in IBD patients were independent of age, sex, and therapeutic drugs for IBD, except the association of depression in IBD patients with the development of Alzheimer’s dementia was greater in patients with DM.

To the best of our knowledge, this is the first study in which the effects of depression on the development of dementia and/or PD in patients with IBD were analyzed. Furthermore, this was the largest population-based cohort study to date in which claims data were used to explain the risk of developing dementia and/or PD in IBD patients with depression. In previous studies, IBD patients were shown to have a high risk of depression, and results of the present study demonstrated the presence of depression in IBD patients can be a risk for neurodegenerative disorders including dementia and PD (14). Consequently, these studies show the association between depression and the development of neurological diseases in IBD patients is identifiable.

Several plausible mechanisms may contribute to increased risk of developing dementia and/or PD in IBD patients with depression. First, psychological stress, such as depression, can induce microglial activation, peripheral cell mediated immune reactions, and oxidative and nitrosative stress (24). IBD can also induce systematic inflammation and altered blood-brain barrier permeability with activation of the central inflammatory response (25). When depression is accompanied by IBD, it may be exacerbated due to their synergistic effects. This situation ultimately may leave patients vulnerable to dementia and/or PD. Second, important inflammatory mediators such as interferon gamma and IL-6, which induce increasing gut permeability termed “leaky gut,” are significantly increased in patients with depression (26, 27). This destruction of the intestinal epithelium causes the translocation of bacteria to cross the gut wall and an increase in blood lipopolysaccharide concentration, leading to systemic inflammation as well as central neuroinflammation (28). In patients with IBD, gut permeability is already increased and the occurrence of depression exacerbates the leaky gut phenomenon, leading to the possibility of developing dementia and/or PD. Third, the alteration of gut microbiota can contribute to developing dementia and/or PD (29). Depression causes changes in gut microbiota, and changes in the composition of specific microbiota, such as increased abundance of *Firmicutes* and reduced abundance of *Proteobacteria*, have been identified in previous studies (30, 31). In addition, these changes in the microbiota were confirmed in patients with Alzheimer’s disease and PD which supports the results of the present study (31, 32).

The results of this study showed depression independently increased the risk of dementia and/or PD regardless of sex and age. In a recent meta-analysis (33), the risk of PD between males and females reportedly did not differ, and in a population-based cohort study in Taiwan (13), significant sex differences for dementia were not found, which is in accordance with the present study results. However, in terms of age and risk, a higher risk of developing PD in older (>65 years of age) IBD patients was reported in a meta-analysis. The results in the present study revealed a similar risk of dementia and/or PD between age subgroups (≤60 vs. >60 years, dementia: HR 2.95 vs. 1.92, PD: HR 0.80 vs. 2.86) in IBD patients with depression. This result is likely because the effect of depression is dominant in the development of dementia and PD compared to the effect of age. Regarding co-morbidities, the effects of depression in IBD patients on the development of Alzheimer’s dementia were significantly greater in patients with DM. In previous studies, DM was shown a risk factor for Alzheimer’s disease (34) and in patients with DM, the severity of depression and IBD may increase (35, 36). Therefore, we postulate the effects of depression on the development of Alzheimer’s dementia in patients with DM may be amplified. However, further studies are needed to analyze the effects of DM as well as other comorbidities on developing Alzheimer’s dementia in IBD with depression.

The present study had several limitations due to its retrospective design. First, the risk of developing dementia and/or PD based on IBD disease severity could not be determined because the disease severity was not available from the HIRA database. To overcome this limitation, analysis based on the use of therapeutic drugs for IBD, including 5-ASA, steroids, and immunomodulators, as indicators of the disease severity was performed and difference in the incidence of dementia and PD was not found. Second, multivariable Cox regression models were used for minimizing confounding effects on developing dementia and/or PD, however, some potential confounding factors, such as lifestyle or personal health behaviors, could not be included. Smoking and alcohol consumption may be associated with developing neurodegenerative disorders as well as IBD, thus, further investigations are required to evaluate the risk of developing dementia and/or PD based on these factors (37, 38). Third, although the results showed that depression in patients with IBD increases the risk of dementia and PD, and we suggested several possible hypotheses, the specific pathophysiology could not be explained. Fourth, statistical significance could not be obtained in some results due to the insufficient number of patients with both CD and depression. Finally, the risk of developing dementia and/or PD among IBD patients based on severity and the treatment response of depression could not be assessed due to the retrospective design. Further analysis is needed using a prospective cohort to elucidate the effect of treatment for depression on developing dementia and/or PD in IBD patients.

In conclusion, the risk of developing dementia and/or PD increased after a diagnosis of depression in patients with IBD. Therefore, if depression is accompanied by IBD, physicians should consider the risk of developing dementia and/or PD may increase.

## Data availability statement

The data used and/or analyzed in this article will be shared upon reasonable request to the corresponding author.

## Ethics statement

The studies involving human participants were reviewed and approved by Seoul National University College of Medicine-Seoul National University Hospital Institutional Review Board. Written informed consent for participation was not required for this study in accordance with the national legislation and the institutional requirements.

## Author contributions

KC: conceptualization, data curation, and writing—original draft preparation. KH: methodology, formal analysis, and resources. KC, HJL, and JK: validation. HJL, JPI, and S-JK: writing—review and editing. KC, KH, HJL, and JPI: visualization. HJL: supervision and project administration. All authors: approval of the final manuscript.

## Footnotes

1
http://www.R-project.org

## Supplementary material

Supplementary Figure 1 The cumulative incidence of Alzheimer’s dementia **(A–C)** and vascular dementia **(D–F)** in inflammatory bowel disease (IBD), Crohn’s disease (CD), and ulcerative colitis (UC) patients with depression compared with those without depression.

Supplementary Figure 2 Subgroup analysis of the risk of developing Alzheimer’s dementia **(A)** and vascular dementia **(B)** based on the presence of depression in patients with IBD. IBD, inflammatory bowel disease; CI, confidence interval.

Supplementary Table 1 Independent risk of Alzheimer’s dementia based on the presence of depression in patients with inflammatory bowel disease (IBD).

Supplementary Table 2 Independent risk of vascular dementia based on the presence of depression in patients with inflammatory bowel disease (IBD).

## Abbreviations

ASA: amino-salicylic acid
CD: Crohn’s disease
CI: confidence interval
DM: diabetes mellitus
aHR: adjusted hazard ratio
IBD: inflammatory bowel disease
IL: interleukin
ICD: international classification of disease
HIRA: health insurance review and assessment service
PD: Parkinson’s disease
RID: rare intractable disease
SD: standard deviation
UC: ulcerative colitis.
