# Supplementary figures and images for: Depression in patients with inflammatory bowel disease is associated with increased risk of dementia and Parkinson’s disease: A nationwide, population-based study
Source: Front Med (Lausanne). 2022 Oct 6;9:1014290. doi: 10.3389/fmed.2022.1014290 (PMC9582438; doi:10.3389/fmed.2022.1014290)

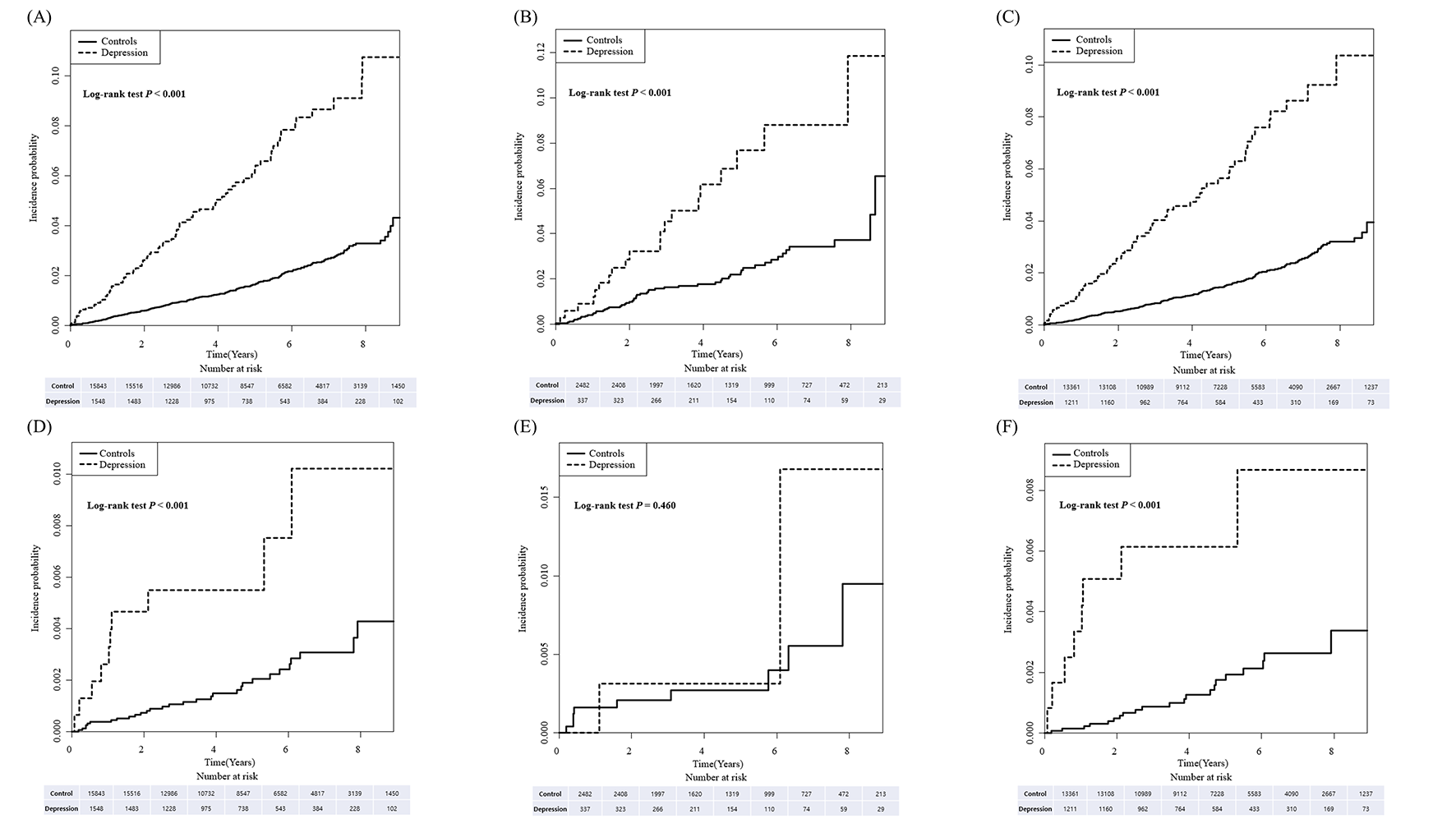

Supplement: Supplementary Figure 1 — The cumulative incidence of Alzheimer’s dementia (A–C) and vascular dementia (D–F) in inflammatory bowel disease (IBD), Crohn’s disease (CD), and ulcerative colitis (UC) patients with depression compared with those without depression. [file Image_1.TIF]

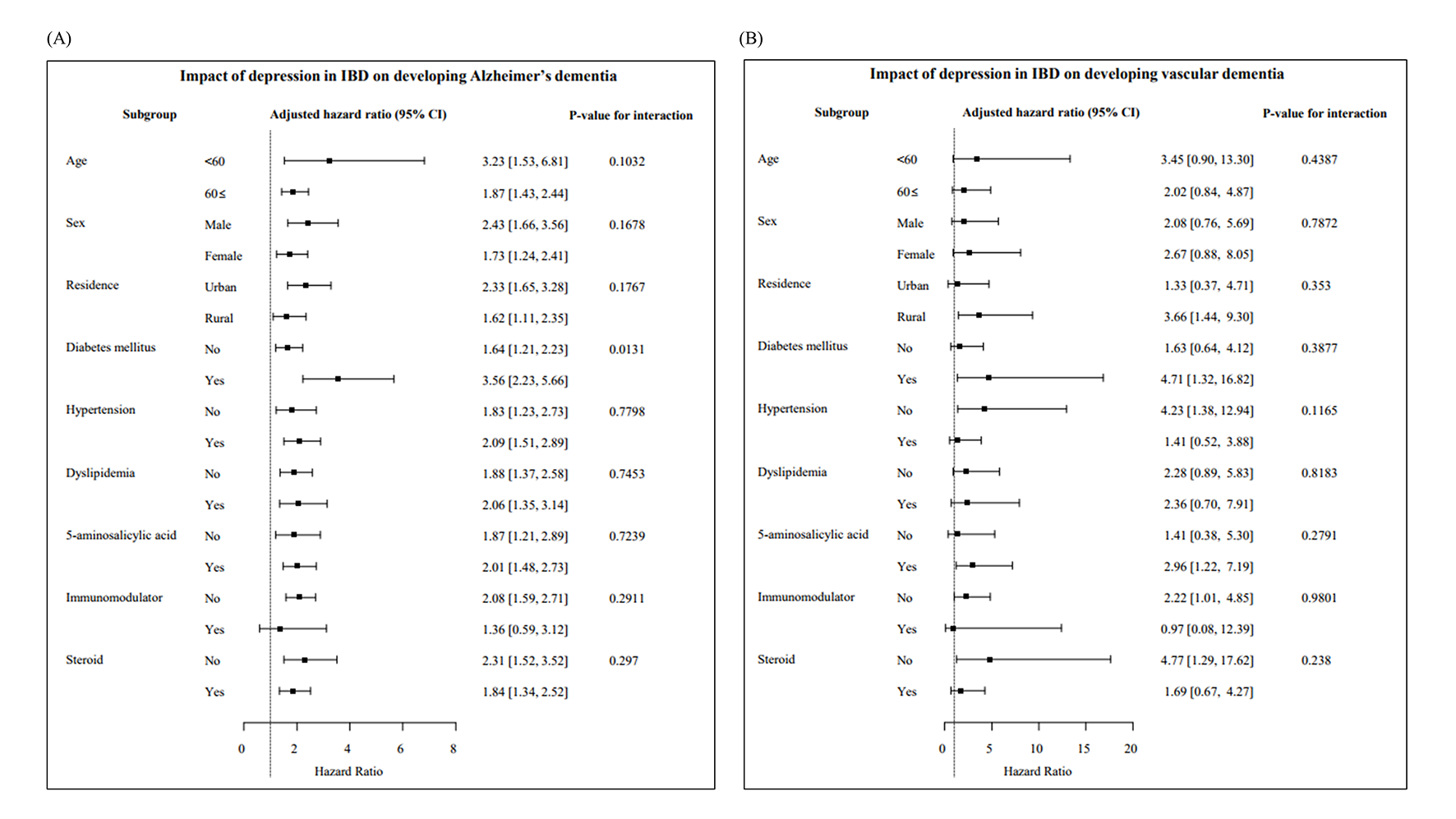

Supplement: Supplementary Figure 2 — Subgroup analysis of the risk of developing Alzheimer’s dementia (A) and vascular dementia (B) based on the presence of depression in patients with IBD. IBD, inflammatory bowel disease; CI, confidence interval. [file Image_2.TIF]
