# Supplementary material for: Depression in patients with inflammatory bowel disease is associated with increased risk of dementia and Parkinson’s disease: A nationwide, population-based study
Source: Front Med (Lausanne). 2022 Oct 6;9:1014290. doi: 10.3389/fmed.2022.1014290 (PMC9582438; doi:10.3389/fmed.2022.1014290)
Supplement: Supplementary Table 1 — Independent risk of Alzheimer’s dementia based on the presence of depression in patients with inflammatory bowel disease (IBD). [file Table_1.DOCX]

**Supplementary Table 1. Independent risk of Alzheimer’s dementia according to presence of depression in patients with IBD**

|  | Total No. | Events (n) | Follow-Up Duration (Person-Years) | Incidence Rate (Per 1000 Person-Years) | Model 1^†^ HR (95% C.L.) | P-value | Model 2^‡^ HR (95% C.L.) | P-value | Model 3^§^ HR (95% C.L.) | P-value |
| --- | --- | --- | --- | --- | --- | --- | --- | --- | --- | --- |
| IBD |  |  |  |  |  | <0.001 |  | <0.001 |  | <0.001 |
| Without depression | 15,843 | 264 | 7,175 | 3.68 | 1(Ref.) |  | 1(Ref.) |  | 1(Ref.) |  |
| With depression | 1,548 | 85 | 6,441 | 13.19 | 2.20(1.72-2.81) |  | 2.05(1.59-2.63) |  | 1.99(1.55-2.56) |  |
| Subgroup |  |  |  |  |  |  |  |  |  |  |
| Incident | 849 | 36 | 3,679 | 9.78 | 1.91(1.35-2.71) | <0.001 | 1.75(1.23-2.48) | 0.002 | 1.68(1.18-2.38) | 0.004 |
| Prevalent | 699 | 49 | 2,762 | 17.73 | 2.48(1.82-3.37) | <0.001 | 2.35(1.72-3.21) | <0.001 | 2.32(1.69-3.17) | <0.001 |
| CD |  |  |  |  |  | 0.012 |  | 0.056 |  | 0.052 |
| Without depression | 2,482 | 55 | 10,978 | 5.01 | 1(Ref.) |  | 1(Ref.) |  | 1(Ref.) |  |
| With depression | 337 | 20 | 1,393 | 14.35 | 1.93(1.15-3.22) |  | 1.67(0.99-2.82) |  | 1.68(0.99-2.85) |  |
| Subgroup |  |  |  |  |  |  |  |  |  |  |
| Incident | 168 | 5 | 754 | 6.63 | 1.25(0.50-3.12) | 0.638 | 1.05(0.42-2.66) | 0.912 | 1.01(0.40-2.56) | 0.980 |
| Prevalent | 169 | 15 | 639 | 23.46 | 2.35(1.33-4.18) | 0.003 | 2.09(1.15-3.80) | 0.015 | 2.18(1.20-3.96) | 0.010 |
| UC |  |  |  |  |  | <0.001 |  | <0.001 |  | <0.001 |
| Without depression | 13,361 | 209 | 60,772 | 3.44 | 1(Ref.) |  | 1(Ref.) |  | 1(Ref.) |  |
| With depression | 1,211 | 65 | 5,048 | 12.88 | 2.25(1.70-2.98) |  | 2.13(1.60-2.83) |  | 2.07(1.56-2.75) |  |
| Subgroup |  |  |  |  |  |  |  |  |  |  |
| Incident | 681 | 31 | 2,925 | 10.60 | 2.10(1.44-3.07) | <0.001 | 1.98(1.35-2.89) | <0.001 | 1.90(1.30-2.79) | <0.001 |
| Prevalent | 530 | 34 | 2,123 | 16.01 | 2.40(1.66-3.46) | <0.001 | 2.30(1.59-3.33) | <0.001 | 2.26(1.56-3.28) | <0.001 |

IBD, Inflammatory bowel disease; CD, Crohn’s disease; UC, Ulcerative colitis

^†^Model 1: adjusted for age, sex. ^‡^Model 2: adjusted for model 1 + residence, diabetes mellitus, hypertension, dyslipidemia, history of myocardial infarction and stroke.

^§^ Model 3: adjusted for model 2 + medication use for IBD (5-Aminosalicylic acid, immunomodulators, steroid, biologics and small molecule)
