# Supplementary material for: Depression in patients with inflammatory bowel disease is associated with increased risk of dementia and Parkinson’s disease: A nationwide, population-based study
Source: Front Med (Lausanne). 2022 Oct 6;9:1014290. doi: 10.3389/fmed.2022.1014290 (PMC9582438; doi:10.3389/fmed.2022.1014290)
Supplement: Supplementary Table 2 — Independent risk of vascular dementia based on the presence of depression in patients with inflammatory bowel disease (IBD). [file Table_2.DOCX]

**Supplementary Table 2. Independent risk of vascular dementia according to presence of depression in patients with IBD**

|  | Total No. | Events (n) | Follow-Up Duration (Person-Years) | Incidence Rate (Per 1000 Person-Years) | Model 1^†^ HR (95% C.L.) | P-value | Model 2^‡^ HR (95% C.L.) | P-value | Model 3^§^ HR (95% C.L.) | P-value |
| --- | --- | --- | --- | --- | --- | --- | --- | --- | --- | --- |
| IBD |  |  |  |  |  | 0.005 |  | 0.015 |  | 0.027 |
| Without depression | 15,843 | 30 | 7,175 | 0.41 | 1(Ref.) |  | 1(Ref.) |  | 1(Ref.) |  |
| With depression | 1,548 | 10 | 6,441 | 1.55 | 2.80(1.35-5.78) |  | 2.47(1.19-5.16) |  | 2.30(1.10-4.82) |  |
| Subgroup |  |  |  |  |  |  |  |  |  |  |
| Incident | 849 | 5 | 3,679 | 1.36 | 2.70(1.04-6.98) | 0.041 | 2.34(0.90-6.09) | 0.081 | 2.18(0.83-5.70) | 0.112 |
| Prevalent | 699 | 5 | 2,762 | 1.81 | 2.91(1.11-7.60) | 0.029 | 2.63(0.99-6.95) | 0.051 | 2.43(0.92-6.44) | 0.074 |
| CD |  |  |  |  |  | 0.710 |  | 0.710 |  | 0.749 |
| Without depression | 2,482 | 9 | 10,978 | 0.82 | 1(Ref.) |  | 1(Ref.) |  | 1(Ref.) |  |
| With depression | 337 | 2 | 1,393 | 1.43 | 1.34(0.29-6.27) |  | 1.34(0.29-6.31) |  | 1.29(0.27-6.06) |  |
| Subgroup |  |  |  |  |  |  |  |  |  |  |
| Incident | 168 | 2 | 754 | 2.65 | 2.93(0.63-13.64) | 0.171 | 2.85(0.61-13.37) | 0.183 | 2.81(0.60-13.22) | 0.190 |
| Prevalent | 169 | 0 | 639 | 0 | - | - | - | - | - | - |
| UC |  |  |  |  |  | 0.004 |  | 0.010 |  | 0.015 |
| Without depression | 13,361 | 21 | 60,772 | 0.34 | 1(Ref.) |  | 1(Ref.) |  | 1(Ref.) |  |
| With depression | 1,211 | 8 | 5,048 | 1.58 | 3.43(1.50-7.84) |  | 3.01(1.30-6.97) |  | 2.86(1.23-6.67) |  |
| Subgroup |  |  |  |  |  |  |  |  |  |  |
| Incident | 681 | 3 | 2,925 | 1.02 | 2.38(0.71-8.03) | 0.162 | 2.12(0.63-7.16) | 0.227 | 2.00(0.59-6.79) | 0.268 |
| Prevalent | 530 | 5 | 2,123 | 2.35 | 4.69(1.73-12.67) | 0.002 | 4.12(1.49-11.37) | 0.006 | 3.95(1.42-10.99) | 0.008 |

IBD, Inflammatory bowel disease; CD, Crohn’s disease; UC, Ulcerative colitis

^†^Model 1: adjusted for age, sex. ^‡^Model 2: adjusted for model 1 + residence, diabetes mellitus, hypertension, dyslipidemia, history of myocardial infarction and stroke.

^§^ Model 3: adjusted for model 2 + medication use for IBD (5-Aminosalicylic acid, immunomodulators, steroid, biologics and small molecule)
